# Construction of a novel mRNA-miRNA-lncRNA network and identification of potential regulatory axis associated with prognosis in colorectal cancer liver metastases

**DOI:** 10.18632/aging.203049

**Published:** 2021-06-03

**Authors:** Chen Lu, Xiagang Luo, Cheng Xing, Yonghuan Mao, Yuting Xu, Wenjie Gao, Wulin Wang, Tian Zhan, Guoguang Wang, Zhengxia Liu, Chunzhao Yu

**Affiliations:** 1 Department of General Surgery, The Second Affiliated Hospital of Nanjing Medical University, Nanjing 210011, Jiangsu, China; 2 Department of Geriatrics, The Second Affiliated Hospital of Nanjing Medical University, Nanjing 210011, Jiangsu, China; 3 Department of Ophthalmology, Zhongnan Hospital of Wuhan University, Wuhan 430071, Hubei, China; 4 Department of Gastrointestinal Surgery, Jingzhou Central Hospital, Jingzhou 434000, Hubei, China

**Keywords:** colorectal cancer liver metastases, prognosis, bioinformatics analysis, competing endogenous RNA (ceRNA)

## Abstract

Liver metastasis is a leading cause of death in patients with colorectal cancer (CRC). Increasing evidence demonstrates that competing endogenous RNA (ceRNA) networks play important roles in malignant cancers. The purpose of this study was to identify molecular markers and build a ceRNA network as a significant predictor of colorectal liver metastases (CRLM). By integrated bioinformatics analysis, we found that apolipoprotein C1 (APOC1) was upregulated in CRLM and associated with prognosis in patients with CRC and thereby established an APOC1-dependent ceRNA network. By survival analysis, expression analysis, and correlation analysis of each element in the ceRNA network, we identified that ZEB1-AS1, miR-335-5p and APOC1 regulated each other. We further experimentally confirmed that ZEB1-AS1 promoted a CRC progression via regulating the expression of miR-335-5p that controlled the expression of APOC1. Our findings indicate that the ZEB1-AS1-miR-335-5p-APOC1 ceRNA regulatory network is significantly valuable for better prognosis of patients with CRC and as a new therapeutic target for the treatment of CRLM.

## INTRODUCTION

Colorectal cancer (CRC) is one of the most common malignant tumors of the digestive tract, and ranks as the second leading cause of cancer-related deaths worldwide [1]. This is despite the fact that the diagnosis and treatment have made great progress in the past decades. CRC metastasis to liver is the main death cause of such patients. Almost 50% of patients with CRC have metastasized to the liver termed as liver metastases, of which approximately 15%-25% of patients have synchronous liver metastases, while 20% have metachronous liver metastases [2, 3]. The median survival time of patients with colorectal liver metastasis (CRLM) without active treatment is only 6-9 months, and the 5-year survival rate of patients with unresectable liver metastases is less than 5% [4, 5]. It is of vital importance to investigate potential molecular mechanisms of CRLM, to find out novel prognostic biomarkers and to identify an effective therapeutic target for colorectal cancer metastases.

Human transcriptome contains protein-coding RNAs and noncoding RNAs. Noncoding RNAs, which encode no proteins, account for over 98% of the entire genome transcripts and can usually be divided into small noncoding RNAs within 200 nucleotides in length and long noncoding RNAs (lncRNAs) over 200 nucleotides in length [6]. In 2011, competing endogenous RNAs (ceRNAs), which regulate other RNA transcripts by competing for shared microRNAs (miRNAs), were further classified [7]. Some lncRNAs had been found to be able to function as ceRNAs and serve as natural miRNA sponges to inhibit miRNA functions by sharing miRNA response elements (MRE), thereby increasing the transcription level of miRNA’s targets [7, 8]. Numerous evidence has well proved that ceRNA networks play important roles in multitudinous cancers, like gastric cancer [9, 10], hepatocellular carcinoma [11, 12], breast cancer [13, 14], colorectal cancer [15, 16] and pancreatic cancer [17].

However, information for ceRNAs associated with prognosis in CRC metastases is very limited. In this study, in order to acquire the differentially expressed genes (DEGs) associated with colorectal cancer metastases, we downloaded 4 gene expression profile datasets (GSE41258, GSE49355, GSE68468 and GSE81558) and 1 miRNA expression profile dataset (GSE35834) from GEO database. We then analyzed DEGs through integrated bioinformatics methods and selected some key genes in CRC metastases for functional enrichment analysis, survival analysis, and expression analysis. In parallel, we predicted upstream noncoding RNAs (including miRNAs and lncRNAs), and evaluated them for prognosis, expression and correlation. We finally demonstrated by *in vitro* experiments that lncRNA ZEB1-AS1 was a miR-335-5p sponge, and regulated APOC1 expression and encouraged CRC cell invasion and migration through interaction with miR-335-5p. Our results revealed for the first time that a novel ceRNA regulatory network was related to prognosis and progression in CRLM. It is expected that the elements involved in the network may have the potential to become clinical biomarkers for prognosis and to be used for therapeutic targets of CRLM in the future.

## RESULTS

### Screening of DEGs

**Figure 1:**
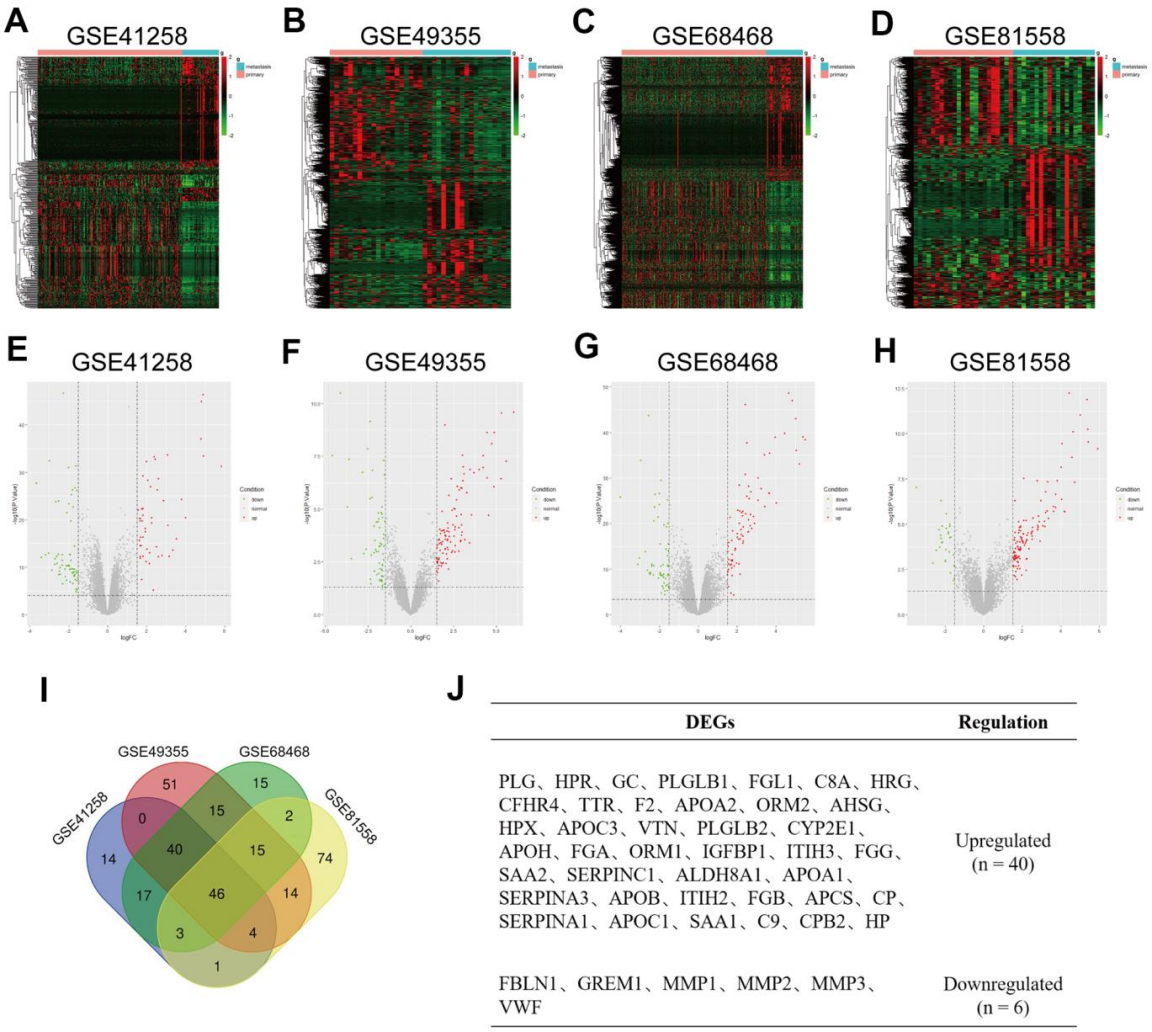
**Identification of differentially expressed genes (DEGs) in colorectal liver metastases (CRLM).** (**A**–**D**) Heatmap of top 500 variable of DEGs between CRC primary tumor tissues and CRC liver metastatic tumor tissues in GSE41258, GSE49355, GSE68468 and GSE81558 datasets. (**E**–**H**) Volcano plot of DEGs identified from GSE41258, GSE49355, GSE68468 and GSE81558 datasets. Green dots express downregulated DEGs, and Red dots represent upregulated DEGs under the same thresholds. The gray dots denote genes that are not differentially expressed. |log2FC| > 1.5 and P-value < 0.05 were set as the cut-off criteria. (**I**) The intersection of DEGs of GSE41258, GSE49355, GSE68468 and GSE81558 datasets. (**J**) List of consistent DEGs, including 40 upregulated DEGs and 6 downregulated DEGs.

Data on primary tumor lesions and liver metastatic tumor lesions were extracted from GSE41258, GSE49355, GSE68468 and GSE81558 datasets. DEGs were screened using P-value < 0.05 and |logFC| >1.5 as cut-off criteria, respectively. Variable DEGs between CRC primary tumor tissues and CRC liver metastatic tumor tissues were identified with ggplot2 and PheatMap packages of R (Figure 1A–1H). With limma packages of R and integrated bioinformatics analysis, 46 DEGs were found to express consistently in four datasets, of which 40 were upregulated while 6 were downregulated (Figure 1I). Names of these regulated DEGs are given in Figure 1J. Their fold changes, P values and other information are listed in Supplementary Table 1.

### Functional enrichment analysis of DEGs

**Figure 2:**
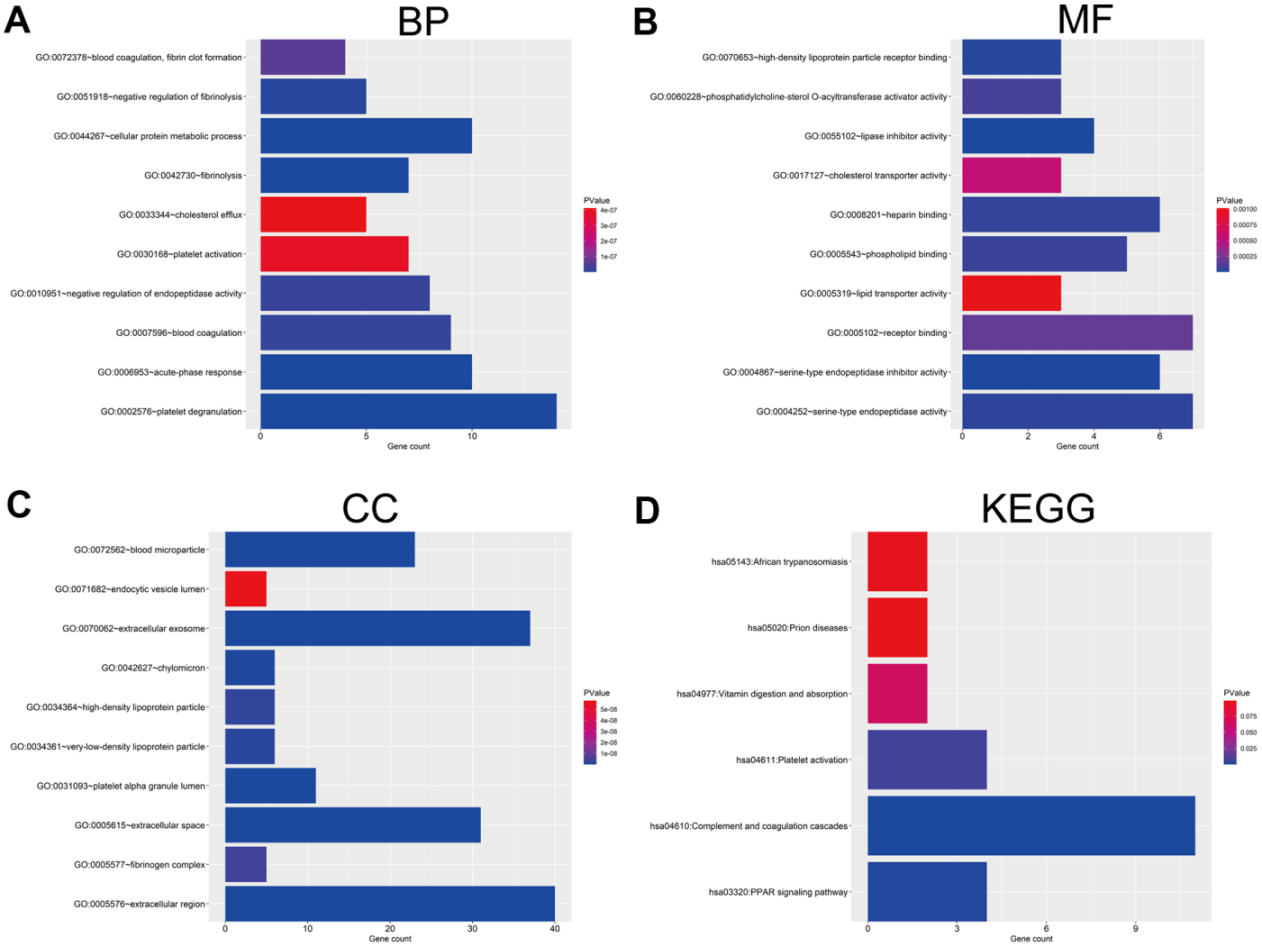
**GO and KEGG pathway enrichment analysis of DEGs.** (**A**) The top 10 enriched biological process (BP) of the 46 DEGs. (**B**) The top 10 enriched molecular function (MF) of the 46 DEGs. (**C**) The top 10 enriched cellular component (CC) of the 46 DEGs. (**D**) The enriched KEGG pathways of the 46 DEGs. GO, Gene Ontology; KEGG, Kyoto Encyclopedia of Genes and Genomes.

We further predicted the potential biological function and relevant pathways of the 46 regulated DEGs using DAVID database that can conduct functional enrichment analysis, including GO functional enrichment and KEGG pathway enrichment analysis. GO functional enrichment analysis contains three aspects, biological process (BP), cellular component (CC) and molecular function (MF). Within the BP aspect, the DEGs were most enriched in ‘platelet degranulation’, ‘acute-phase response’, ‘fibrinolysis’, ‘cellular protein metabolic process’, ‘negative regulation of fibrinolysis’, ‘blood coagulation’, ‘negative regulation of endopeptidase activity’, ‘blood coagulation, fibrin clot formation’, ‘platelet activation’ and ‘cholesterol efflux’ (Figure 2A). Within the CC aspect, the DEGs were primarily enriched in ‘cholesterol efflux’, ‘blood microparticle’, ‘extracellular space’, ‘extracellular exosome’, ‘platelet alpha granule lumen’, ‘chylomicron’, ‘very-low-density lipoprotein particle’, ‘high-density lipoprotein particle’, ‘fibrinogen complex’ and ‘endocytic vesicle lumen’ (Figure 2B). And within the MF aspect, the DEGs were most enrich in ‘endocytic vesicle lumen’, ‘serine-type endopeptidase inhibitor activity’, ‘high-density lipoprotein particle receptor binding’, ‘serine-type endopeptidase activity’, ‘heparin binding’, ‘phospholipid binding’, ‘phosphatidylcholine-sterol O-acyltransferase activator activity’, ‘receptor binding’, ‘cholesterol transporter activity’ and ‘lipid transporter activity’ (Figure 2C). In the KEGG pathway category, the DEGs were enriched in ‘PPAR signaling pathway’, ‘complement and coagulation cascades’ and ‘platelet activation’ (Figure 2D).

### Survival analysis and expression analysis of key DEGs

**Figure 3:**
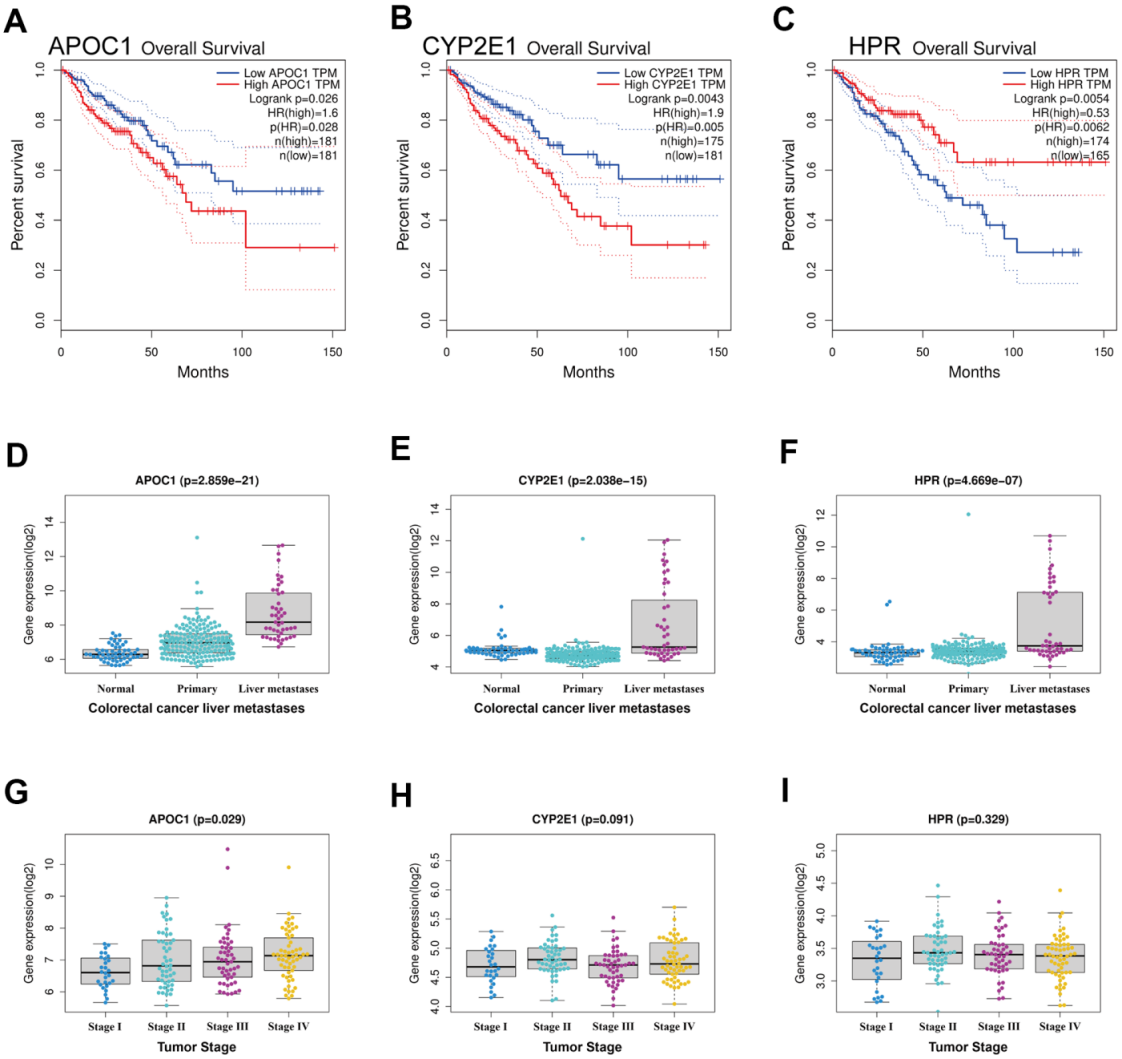
**Survival analysis and expression analysis of the DEGs.** (**A**–**C**) Survival analysis of DEGs in colorectal cancer by using GEPIA database, and only APOC1, CYP2E1 and HPR were significant in term of prognosis. (**D**–**F**) Expression analysis of the three genes (APOC1, CYP2E1 and HPR) between normal tissues, primary tumor tissues and liver metastatic tumor tissues in CRLM by using GSE41258 datasets. (**G**–**I**) Expression analysis grouped by tumor stage of the three genes (APOC1, CYP2E1 and HPR) in CRLM by using GSE41258 datasets.

To obtain key DEGs that could be related to prognosis, the 46 regulated DEGs were applied for survival analysis using GEPIA database. Only three upregulated genes (APOC1, CYP2E1, HPR) were significant in term of prognosis (Figure 3A–3C), suggesting that these three genes could be associated with prognosis in colorectal cancer patients. Expression analysis showed that these three genes highly expressed in CRC liver metastatic tumor lesions, but not in colon normal tissues and CRC primary tumor lesions (Figure 3D–3F). It appeared that APOC1 expressed at the highest level, followed by CYP2E1 and HPR in all tumor stages (Figure 3G–3I).

### APOC1 is targeted by hsa-miR-335-5p

**Figure 4:**
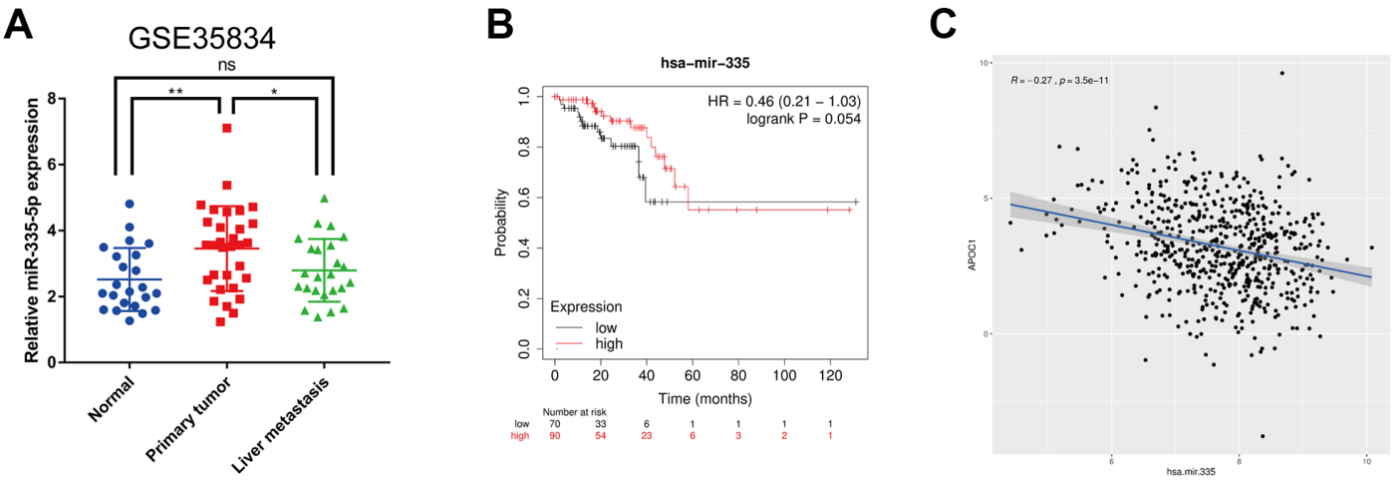
**Expression analysis, survival analysis and correlation analysis of hsa-miR-335-5p.** (**A**) Expression analysis of hsa-miR-335-5p among normal tissues, primary tumor tissues and liver metastatic tumor tissues in CRLM using GSE35834 datasets. (**B**) Survival analysis of hsa-miR-335 by using Kaplan-Meier plotter database. (**C**) Correlation analysis of hsa-miR-335 and APOC1 in TCGA project by using ggplot2 and ggpubr packages of R. ns (Not significant), *p < 0.05, **p < 0.01.

By using the miRTarbase database hsa-miR-335-5p was found to be the only miRNA that could potentially regulate APOC1 in the database. To see this miRNA expression in CRC, a miRNA expression profile dataset (GSE35834) associated with CRLM was downloaded from the GEO database. Comparative analyses showed that the expression of miR-335-5p was up-regulated in CRC tumor tissues but its expression was higher in primary tumor lesions than in liver metastatic tumor lesions (Figure 4A). Survival analysis using the Kaplan-Meier plotter database and correlation analysis using the ggplot2 and ggpubr packages of R and the method of “Pearson” showed that although hsa-miR-335-5p had no statistical significance in prognosis (P = 0.054), its low expression resulted in low survival rate compared to its high expression, which resulted in high survival rate (HR = 0.46) (Figure 4B). Correlation analysis revealed that APOC1 was negatively regulated by hsa-miR-335-5p in colorectal cancer (r = -0.27, P < 0.001) (Figure 4C).

### Bioinformatic identification of lncRNAs binding hsa-miR-335-5p

**Figure 5:**
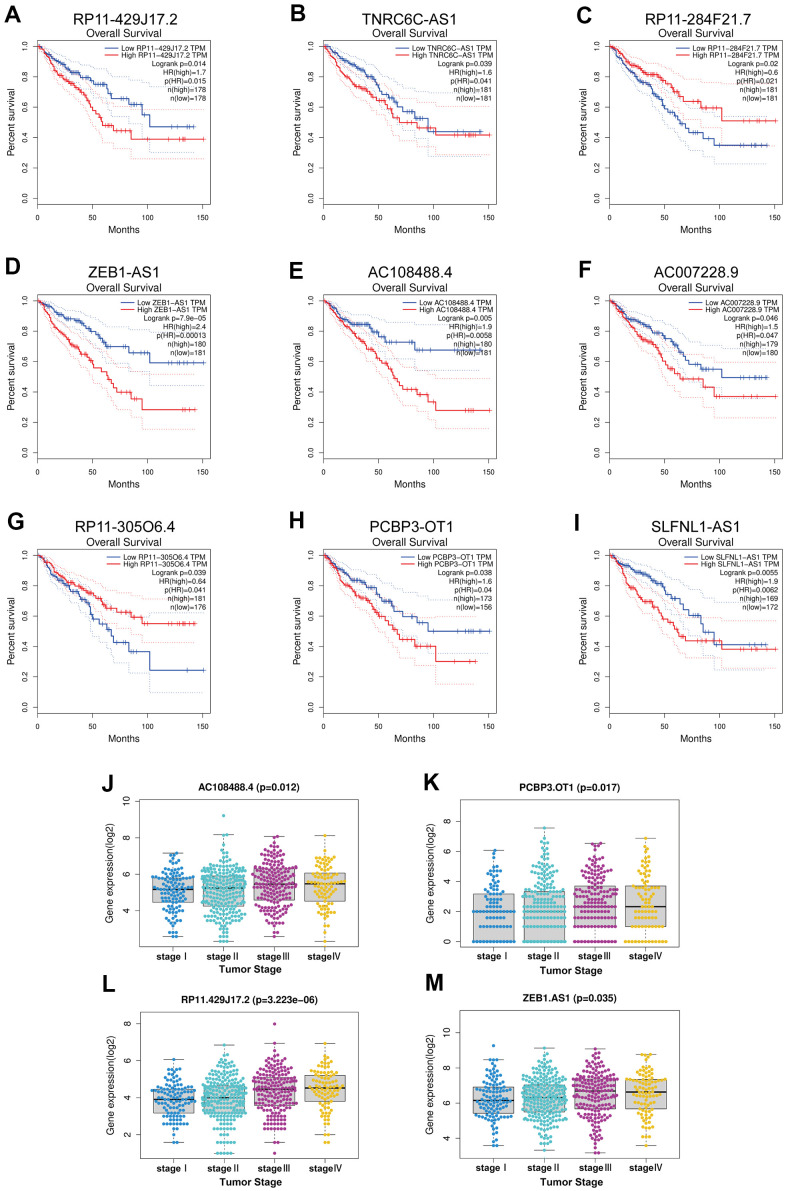
**Survival analysis and expression analysis of upstream key lncRNAs.** (**A**–**I**) Prognostic value of the nine lncRNAs (RP11-429J17.2, TNRC6C-AS1, RP11-284F21.7, ZEB1-AS1, AC108488.4, AC007228.9, RP11-305O6.4, PCBP3-OT1, SLFNL1-AS1) in CRC by using GEPIA database. (**J**–**M**) Expression analysis grouped by tumor stage of the four lncRNAs (RP11-429J17.2, AC108488.4, PCBP3-OT1, ZEB1-AS1) in CRC by using TCGA project.

As previously described, lncRNAs can inhibit miRNA functions by sharing miRNA response elements [7, 8]. By using the StarBase database,83 lncRNAs were found to have the potential to bind hsa-miR-335-5p (Supplementary Table 2). Survival analysis in colorectal cancer with the GEPIA database showed that of these 83 lncRNAs, only 9 lncRNAs (RP11-429J17.2, TNRC6C-AS1, RP11-284F21.7, ZEB1-AS1, AC108488.4, AC007228.9, RP11-305O6.4, PCBP3-OT1, SLFNL1-AS1) had statistical significance in prognosis of colorectal cancer (Figure 5A–5I). Due to the lack of lncRNA expression information in colorectal cancer liver metastatic tumor tissues and in other metastatic tumor tissues, the expression of 9 lncRNAs were analyzed at different tumor stages. Of these 9 lncRNAs, 4 (RP11-429J17.2, ZEB1-AS1, AC108488.4, PCBP3-OT1) had statistical significance in differential expression at different tumor stages (Figure 5J–5M).

### Correlation among lncRNAs, APOC1 and hsa-miR-335-5p

**Figure 6:**
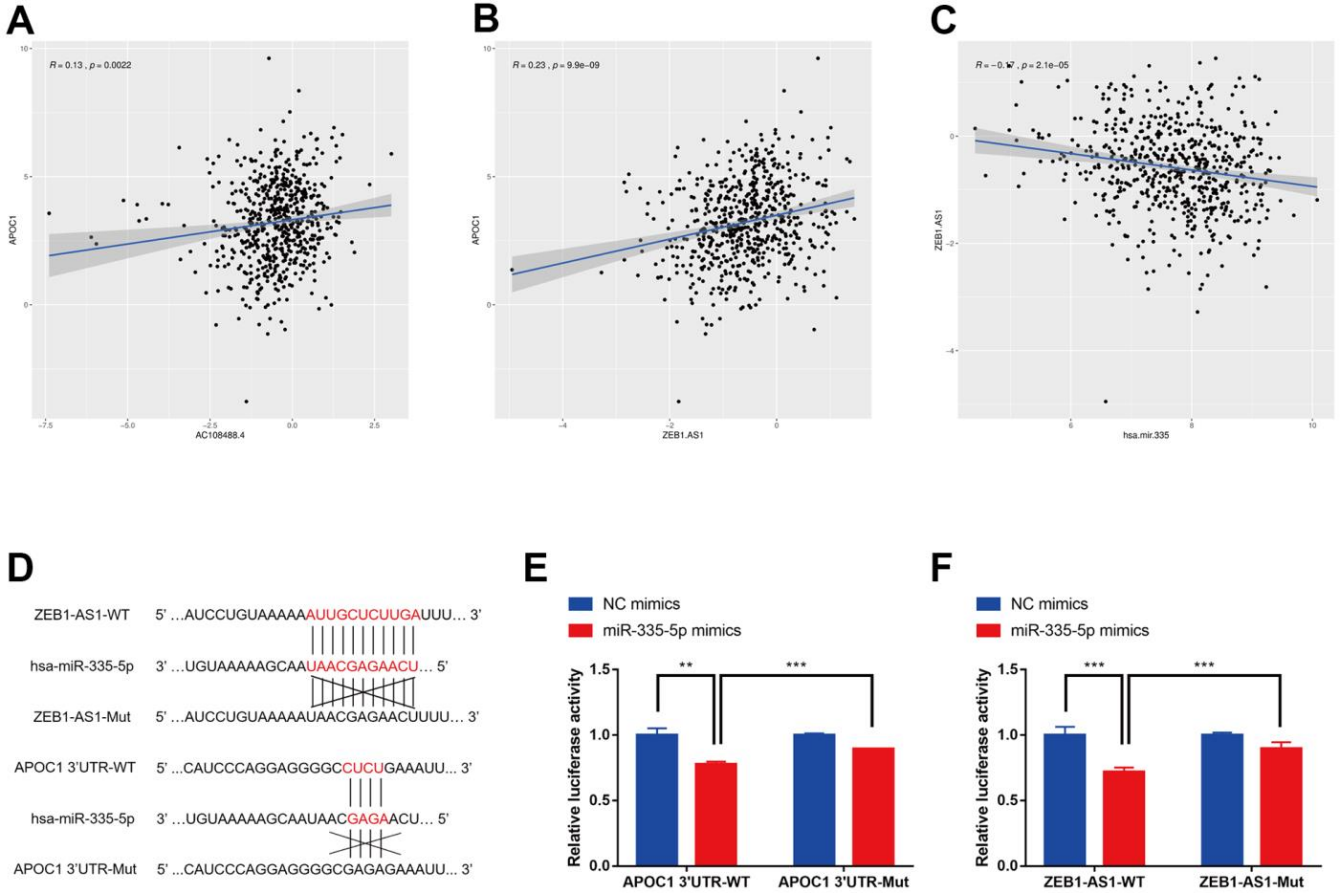
**Correlation analysis among lncRNAs, APOC1 and hsa-miR-335-5p.** (**A**) Correlation analysis between AC108488.4 and APOC1 in TCGA project. (**B**) Correlation analysis between ZEB1-AS1 and APOC1 in TCGA project. (**C**) Correlation analysis between ZEB1-AS1 and hsa-miR-335 in TCGA project. (**D**) Predicted binding sites in ZEB1-AS1 and APOC1 for miR-335-5p. (**E**) Luciferase reporter assay in HCT116 cells co-transfected with wide type (WT) or mutated (Mut) APOC1 3'-UTR reporter vector and miR-335-5p mimics or NC mimics. (**F**) Luciferase reporter assay in HCT116 cells co-transfected with wide type (WT) or mutated (Mut) ZEB1-AS1 reporter vector and miR-335-5p mimics or NC mimics. *p < 0.05, **p < 0.01, *** p < 0.001.

**Figure 7:**
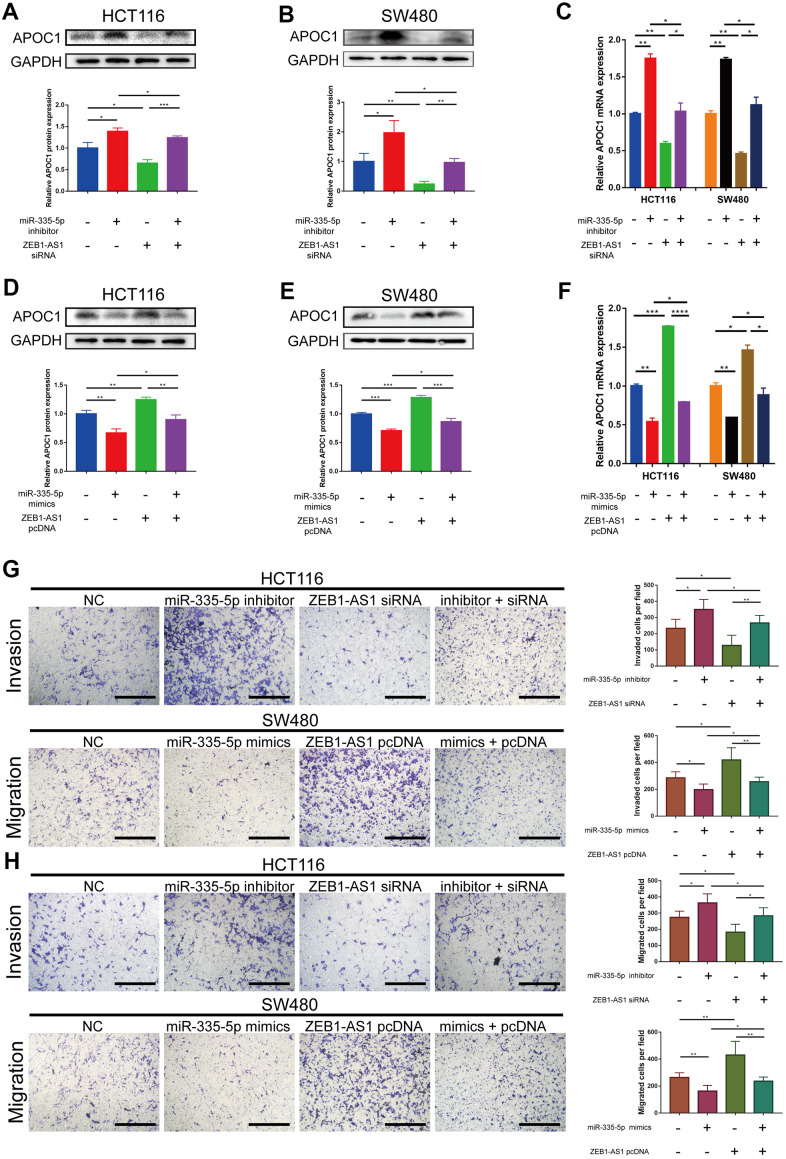
**ZEB1-AS1 regulates APOC1 expression by competing for miR-335-5p to promote CRC cells invasion and migration.** (**A**–**C**) miR-335-5p inhibitor abolished effects of ZEB1-AS1 downregulation on APOC1 expression in HCT116 and SW480 cells. All expression levels were detected by western blotting and RT-qPCR. Representative immunoblots and the ratios of the indicated proteins to GAPDH are presented. (**D**–**F**) ZEB1-AS1 overexpression eliminated the effects of miR-335-5p mimics on APOC1 expression in HCT116 and SW480 cells. All expression levels were detected by western blotting and RT-qPCR. Representative immunoblots and the ratios of the indicated proteins to GAPDH are presented. (**G**, **H**) miR-335-5p inhibitor rescued the effects of ZEB1-AS1 downregulation on HCT116 cell invasion and migration, and ZEB1-AS1 overexpression rescued the effects of miR-335-5p mimics on SW480 cell invasion and migration (Scale bar: 100 μm). *p < 0.05. **p < 0.01. ***p < 0.001. ****p < 0.0001.

The four lncRNAs (RP11-429J17.2, ZEB1-AS1, AC108488.4, PCBP3-OT1) were chosen for analyzing their correlation with APOC1 and hsa-miR-335-5p. By applying the ggplot2 and ggpubr packages of R, AC108488.4 and ZEB1-AS1 were found to be significant in positively regulating APOC1 gene (Figure 6A, 6B. AC108488.4: r = 0.13, P = 0.0022; ZEB1-AS1: r = 0.23, P < 0.001). ZEB1-AS1 had statistical significance in negatively regulating hsa-miR-335-5p, but the others had not (Figure 6C. r = -0.17, P < 0.001). With the StarBase database the potential binding sites between miR-335-5p and ZEB1-AS1 were predicted, while with the miRTarbase database the potential binding sites between miR-335-5p and APOC1 were predicted (Figure 6D). To experimentally confirm the relationship among ZEB1-AS1, miR-335-5p and APOC1, dual-luciferase reporter assays were applied. The result showed that miR-335-5p mimics diminished the relative luciferase activities of ZEB1-AS1-WT and APOC1-WT, but their relative luciferase activities can be restored when the binding site was mutated artificially (Figure 6E, 6F). The effect of ZEB1-AS1 and miR-335-5p on APOC1 expression in cells of HCT116 and SW480 were further confirmed with miR-335-5p inhibitor, miR-335-5p mimics, inhibitor NC, mimics NC, si-ZEB1-AS1, si-NC, pcDNA-ZEB1-AS1 or pcDNA-NC. On the basis of the APOC1 mRNA and protein levels down-regulated ZEB1-AS1 resulted in low expression of APOC1, while down-regulated miR-335-5p increased APOC1 expression. By contrast, ectopic expression of miR-335-5p significantly decreased APOC1 expression, while the overexpression of ZEB1-AS1 increased APOC1 expression (Figure 7A–7F). *In vitro* functionality assays showed that downregulated ZEB1-AS1 reduced HCT116 cell invasion and migration while downregulated miR-335-5p reversed such reduction. Moreover, ectopic expression of miR-335-5p significantly reduced SW480 cell invasion and migration while overexpressed ZEB1-AS1 did them oppositely (Figure 7G, 7H).

### Expression of ZEB1-AS1, miR-335-5p and APOC1 in CRC tumor tissues with metastasis and non-metastasis

**Figure 8:**
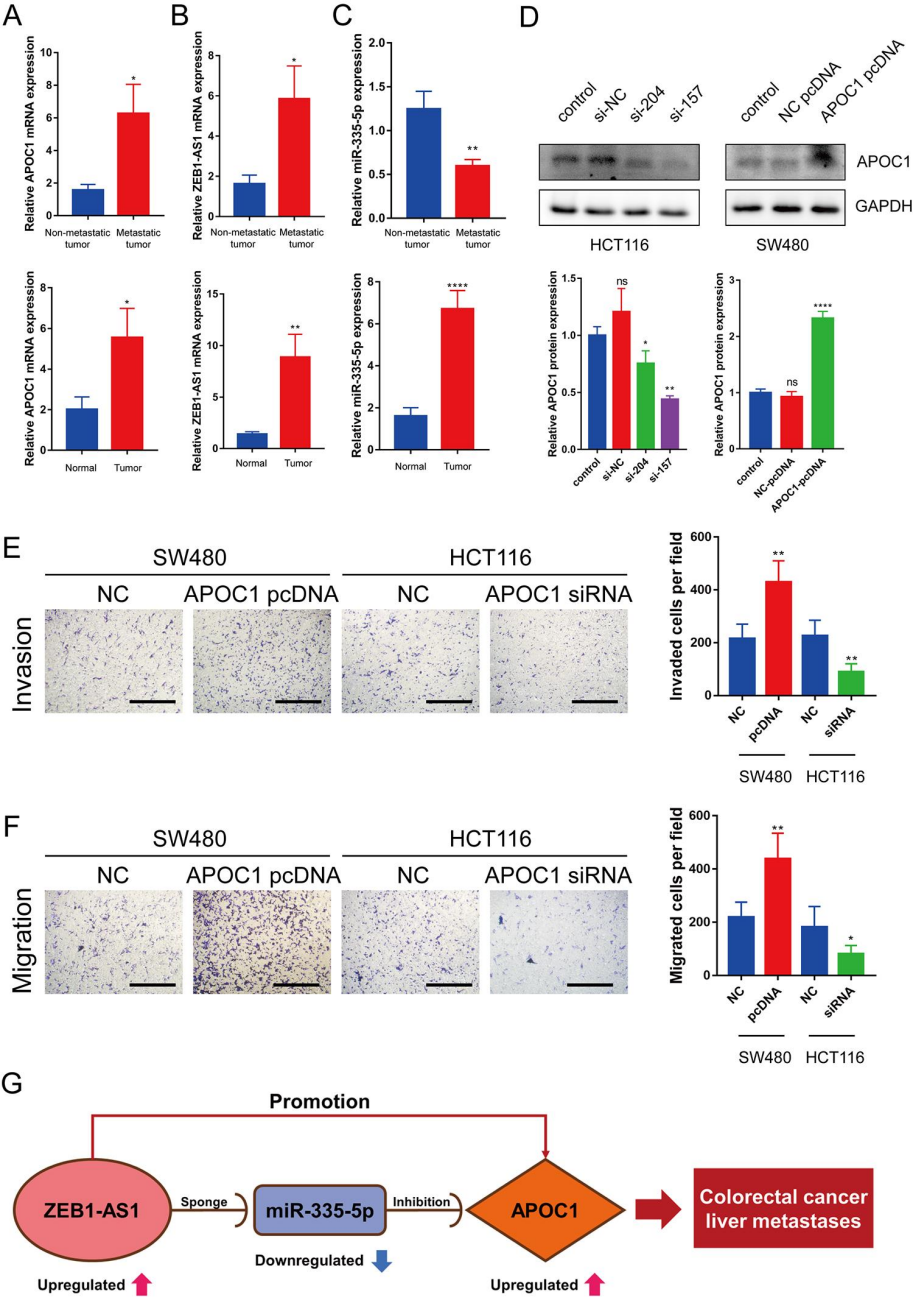
**Expression of ZEB1-AS1, miR-335-5p and APOC1 in CRC tumor tissues and APOC1 promotes CRC cell migration and invasion *in vitro*.** (**A**–**C**) Expression of APOC1, ZEB1-AS1 and miR-335-5p in normal colon tissues and CRC tumor tissues with metastasis or without metastasis were detected by RT-qPCR. (**D**) APOC1 expression was knocked down successfully using small interference RNA (siRNA) in HCT116 cells, and APOC1 expression was overexpressed using a plasmid vector in SW480 cells. All expression levels were detected by western blotting. Representative immunoblots and the ratios of the indicated proteins to GAPDH are presented. (**E**, **F**) Knockdown of APOC1 expression inhibited HCT116 cells migration and invasion, while overexpression of APOC1 increased SW480 cells migration and invasion (Scale bar: 100 μm). (**G**) The novel mRNA-miRNA-lncRNA competing endogenous RNA (ceRNA) triple regulatory sub-network related to prognosis of CRLM. ns (Not significant), *p < 0.05, **p < 0.01, ****p < 0.0001.

To detect the expression of ZEB1-AS1, miR-335-5p and APOC1 in colorectal cancer metastases, RT-qPCR was performed in 30 colorectal cancer tissue samples (16 with metastasis and 14 without metastasis at the time of diagnosis) with adjacent normal tissue samples as a control. APOC1 expression was significantly upregulated in CRC tumor tissues, and was higher in primary tumor lesions with metastatic colorectal cancer than that in the primary tumor lesions without metastatic colorectal cancer (Figure 8A). ZEB1-AS1 expression in CRC tumor tissues was similar to APOC1 expression (Figure 8B), and miR-335-5p expression was up-regulated in CRC tumor tissues but its expression was down-regulated in CRC with metastasis (Figure 8C).

### APOC1 promotes CRC cell migration and invasion *in vitro*

To investigate effects of APOC1 on CRC metastasis, APOC1 was knocked down in HCT116 cells and overexpressed in SW480 cells (Figure 8D). Transwell assays showed that the knockdown of APOC1 expression inhibited HCT116 cells migration and invasion, while the overexpression of APOC1 increased SW480 cells migration and invasion (Figure 8E, 8F). This indicated that APOC1 promoted CRC cell migration and invasion *in vitro*. Taken together, these results indicate that lncRNAs such as ZEB1-AS1 can act as ceRNAs to indirectly regulate mRNAs such as APOC1 through shared miRNAs such as miR-335-5p as previously discovered [18–20]. More specifically, ZEB1-AS1 can promote CRC progression via the miR-335-5p/APOC1 axis. The interaction network among ZEB1-AS1, miR-335-5p and APOC1 is displayed in Figure 8G.

## DISCUSSION

Increasing evidence has indicated that ceRNA networks play a crucial role in tumor progression, metastasis and prognosis [21]. Recently, a novel ceRNA regulatory network (lncRNA MALAT1/miR-129-5p/NFAT5 axis) was discovered to connect to the progression of CRC [22]. However, in term of CRC metastases, only small number of studies was found to be associated with ceRNA and prognosis. Chen et al. found that lncRNA UICLM promoted tumor cell growth, invasion, metastasis and epithelial-mesenchymal transition (EMT) through upregulating ZEB2 expression via miR-215 [23]. Wang et al. demonstrated that USP3 upregulated SMAD4 by competitively binding miR-224, thereby inhibiting CRC cell metastasis [24]. Dong et al. revealed that colorectal cancer progression and metastasis were promoted by lncRNA MIR4435-2HG that upregulates the expression of YAP1 by sponging miR-206 in CRC [25]. Yan et al. showed that a novel lncRNA LINC00483 facilitated colorectal cancer cells metastasis and proliferation by competitively binding miR-204-3p to modulate FMLN2 [26]. However, all of these studies were performed using the gene expression microarray of CRC primary tumor tissues, which is away from the fact that resection of liver metastases was considered to be the only way to cure CRLM patients, and reduction of the volume of liver metastases was the best way to achieve the purpose of surgery [27]. Therefore, exploring a specific ceRNA network like mRNA-miRNA-lncRNA in CRLM would be helpful in the above regards [17].

In the present study, a total of 46 significant DEGs consisting of 40 upregulated genes and 6 downregulated genes were identified by intersection of DEGs from 4 GEO datasets, GSE41258, GSE49355, GSE68468 and GSE81558. Gene ontology analysis showed that most genes were enriched in substances in the hematologic system, including platelet degranulation, fibrinolysis, negative regulation of fibrinolysis, blood coagulation, platelet activation and so on, which may be associated with the route of hematogenous metastasis of CRLM. Lipid metabolism was found to be involved in the occurrence and development of CRLM [28]. Wang et al. discovered that the rate-limiting enzyme CPT1A mediated the fatty acid oxidation for promoting CRC metastases via inhibiting anoikis. This suggests that CPT1A could be a therapeutic target for CRC metastases [29]. Analysis of KEGG pathway enrichment indicated that multiple genes were enriched in “complement and coagulation cascades”, “PPAR signaling pathway” and “platelet activation”. In recent years, increasing evidence revealed that complement and coagulation cascades were involved in tumor cell growth, tumor angiogenesis and immune suppression [30–32]. More studies demonstrated that PPAR promotes inflammation and tumorigenesis [33]. Complement and PPAR signaling were found to be enriched in CRLM via bioinformatics analysis [34]. The interaction of tumor cells with platelets was discovered to be a prerequisite for hematogenous metastasis of the tumor [35].

By using expression and survival analysis of all DEGs in this study APOC1 gene was identified to be important in CRLM. APOC1 gene belongs to the apolipoprotein C family and is primarily synthesized in the liver [36]. It is the smallest of all apolipoproteins and involved in lipid transport and metabolism [37]. Increasing evidence demonstrated that APOC1 gene promotes tumor progression and tumor cell migration [36, 38–41]. Ko et al. discovered that the expression of APOC1 gradually grew from stage I to stage IV in patients with lung cancer [38]. This result is similar to what we observed in CRC. We also found that the expression of APOC1 gradually increased from normal tissues to CRC primary tumor tissues and to CRC liver metastatic tumor tissues. Yi et al. revealed that the concentration of APOC1 in serum was significantly higher in patients with gastric cancer than in healthy individuals. This expression profile is meaningful in relation to tumor stage, lymph node metastasis, tumor classification and survival rate [36]. Ren et al. demonstrated that APOC1 gene promotes tumor progression in CRC through MAPK signaling pathways [39]. This gene is also associated with other types of cancer, like papillary thyroid carcinoma [40] and prostate cancer [41]. However, none of the previous studies defined functional mechanisms of APOC1 in CRLM. According to our results, we propose for the first time that APOC1 could play a crucial role in liver metastasis of colorectal cancer.

We utilized the miRTarbase database to predict miRNAs that target APOC1 and found that miR-335-5p was the only candidate. However, this miRNA had no statistically significance in prognosis when the Kaplan-Meier plotter database was adopted. Due to the lack of data on colon cancer in the Kaplan-Meier plotter database, we could not perform accurate survival analysis. Nevertheless, the correlation analysis revealed that APOC1 gene was negatively regulated by miR-335-5p in colorectal cancer. The suppression effect of miR-335-5p in most tumors had been previously reported [42, 43]. In thyroid cancer, miR-335-5p could interfere with invasion and metastasis of tumor cells via downregulating ICAM-1 [42]. Zhang et al. found that miR-335-5p hindered proliferation, migration and invasion of tumor cells in CRC by downregulating LDHB [43]. Wang et al. discovered that the expression of miR-335-5p in serum was associated with tumor stage and metastasis in CRC [44]. Both studies demonstrated the inhibitory effect of miR-335-5p in CRC, which is consistent with our present result. However, it is worth pointing out that differences in target genes between their studies and ours exist.

Of the 83 predicted lncRNAs, only one lncRNA (ZEB1-AS1) was defined to be the key lncRNA via combining survival analysis, expression analysis and correlation analysis using the GEPIA database and the TCGA database. ZEB1-AS1 has been reported in most tumors [45]. In 2015, Li et al. firstly discovered ZEB1-AS1 to be mainly located in the nucleus and the most efficient lncRNA to promote cellular proliferation in hepatocellular carcinoma [46]. In CRC, ZEB1-AS1 is not only highly expressed in tumor tissues but also associated with poor prognosis and TNM stage [47]. This is consistent with our result in the present study. Other studies also confirmed that ZEB1-AS1 can sponge a few miRNAs to facilitate colorectal cancer cell proliferation, like miR-185a-5p [48], miR-141-3p [49], miR-455-3p [50], miR-205 [51] and miR-101 [52]. Apart from the above, Zhang et al. discovered that lncRNA ZEB1-AS1 promotes tumor proliferation and invasion by downregulating miR-335-5p in gastric cancer [53]. Dual-luciferase reporter assay in this study confirmed the binding of miR-335-5p to ZEB1-AS1 and APOC1 in CRC. Based on the results from Western blot analysis and RT-qPCR, it is very likely that lncRNA ZEB1-AS1 regulates APOC1 by sponging miR-335-5p in CRC. Other functionality experiments indicated that ZEB1-AS1 could sponge miR-335-5p to facilitate cell invasion and migration in CRC.

Overall, our study also has some limitations. Firstly, the expression of lncRNA ZEB1-AS1 in liver metastatic tumor lesions is unknown due to the limitation of the data sources and the difficulty of sample collection. Secondly, we were unable to use animal models to verify the novel ceRNA network. Finally, we only analyzed the expression level of APOC1 and its functions in the ceRNA network, and thus do not know its precise role in CRLM.

In summary, a novel mRNA-miRNA-lncRNA regulatory network was established through integrated bioinformatics analysis and *in vitro* experiments. lncRNA ZEB1-AS1 and APOC1 gene in this network had significant value for prognosis of patients with CRC. The network could provide important insights for molecular mechanisms of CRC metastases and make great contributions to clinical therapy.

## MATERIALS AND METHODS

### Microarray data and clinical data

**Figure 9:**
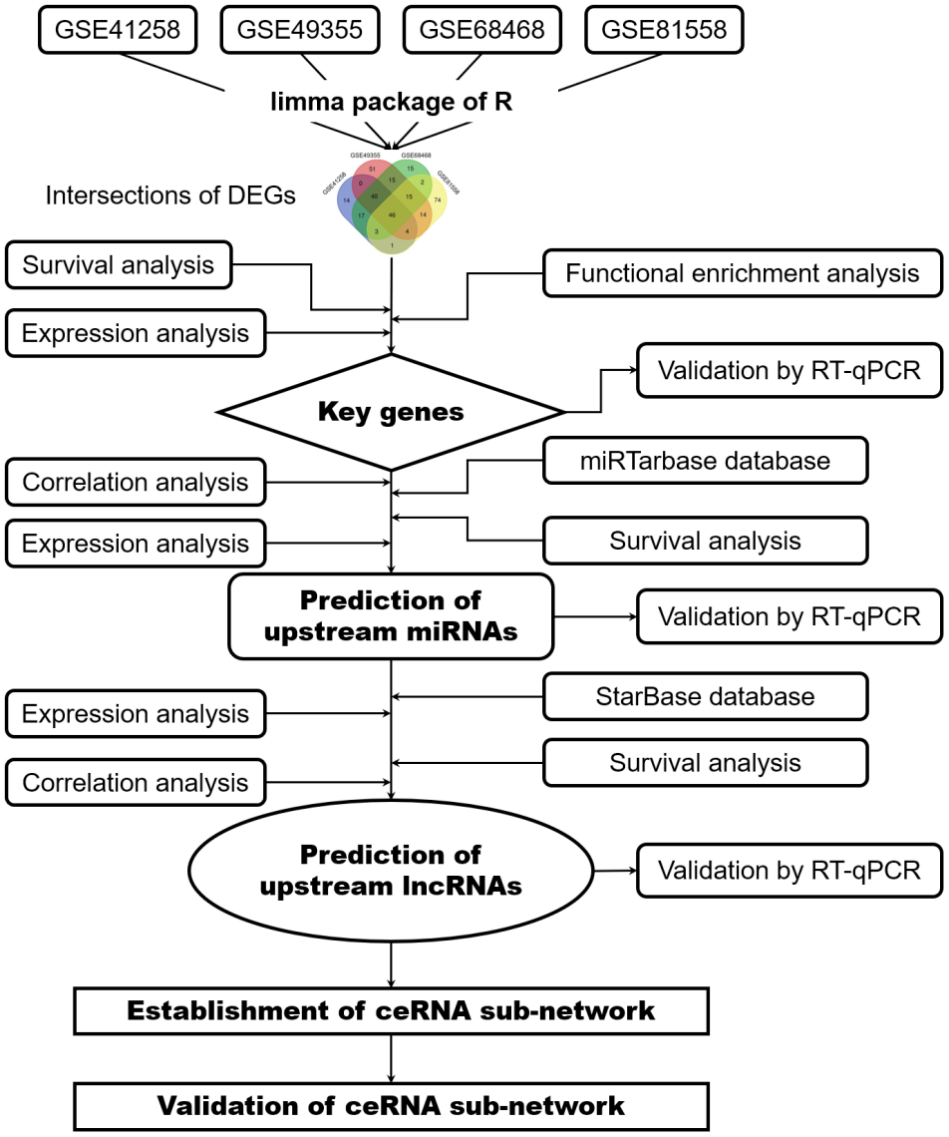
**Flowchart of bioinformatics analysis.** DEGs, differentially expressed genes; PPI network, protein-protein interaction network; miRNAs, microRNAs; lncRNAs, long non-coding RNAs; ceRNA, competing endogenous RNAs.

The procedure of the bioinformatics analysis is presented in a flow diagram (Figure 9). Five datasets (GSE35834, GSE41258, GSE49355, GSE68468 and GSE81558) were downloaded from GEO database (http://www.ncbi.nlm.nih.gov/geo). GSE35834 is a miRNA expression profile related to colon cancer and metastasis development and analyzed based on the GPL8786 platform ([miRNA-1] Affymetrix Multispecies miRNA-1 Array). Three datasets (GSE41258, GSE68468 and GSE49355) were all analyzed based on the GPL96 platform ([HG-U133A] Affymetrix Human Genome U133A Array), while GSE81558 dataset was analyzed based on the GPL15207 platform ([Prime View] Affymetrix Human Gene Expression Array). Primary lesions and liver metastatic lesions of colorectal cancer were selected from the five microarray datasets. Two datasets (GSE41258 and GSE68468) included 186 primary lesions and 47 liver metastatic lesions of CRC. GSE49355 dataset contained 20 primary tumor lesions and 19 liver metastatic tumor lesions. GSE81558 dataset included 23 primary tumor tissues and 19 liver metastatic tumor tissues in colorectal cancer, while GSE35834 dataset included 31 primitive colorectal cancer and 24 liver metastatic samples. Clinical data was obtained from GSE41258 dataset and TCGA database (https://portal.gdc.cancer.gov/), including age, sex, TNM stage and survival information.

### Identification of DEGs

The limma package of R was used to detect DEGs between primary tumor and liver metastatic tumor tissues from the four datasets in colorectal cancer. P-value < 0.05 and |log_2_FC| > 1.5 were set as the cut-off criteria. The Venn webtools (http://bioinformatics.psb.ugent.be/webtools/Venn/) was employed to screen the common genes in all of the four datasets and draw the Venn diagram.

### Functional enrichment analysis of DEGs

The online DAVID webtool (https://david.ncifcrf.gov/) was used to annotate the potential biological process of DEGs, including Gene Ontology (GO) functional enrichment analysis and Kyoto Encyclopedia of Genes and Genomes (KEGG) pathway enrichment analysis [54–56]. P-value < 0.05 was considered as the threshold of the analysis. ggplot2 package of R was used to display the top 10 enriched GO terms and KEGG pathways.

### Prediction of miRNAs

The miRTarbase database was employed to predict miRNAs that target crucial genes [57]. This is one miRNA-related database which collects miRNA-target interactions supported by experimental validation.

### Prediction of lncRNAs

The upstream lncRNAs that bind miRNAs were predicted by utilizing the online database StarBase (https://web.archive.org/web/20110222111721/http://starbase.sysu.edu.cn/). This database can provide information about the interactions between miRNAs and other RNAs from multi-dimensional sequencing data [58].

### Construction of the ceRNA network

We used the Cytoscape software (Version 3.7.2) to establish the ceRNA network based on the ceRNA theory and the associations among lncRNAs, miRNAs and mRNAs.

### Survival analysis

The GEPIA database (http://gepia.cancer-pku.cn/) was employed to analyze prognosis of patients with colorectal cancer. This is a free database for all users and can provide a series of custom functions [59]. Prognostic values of miRNAs were analyzed with the Kaplan-Meier plotter database (http://kmplot.com/analysis/), which can evaluate the impact of over 50 thousand genes on the survival of 21 cancer categories [60]. In the Kaplan-Meier plotter database, only “Rectum adenocarcinoma” item was selected. Both “COAD” and “READ” items were chosen in the GEPIA database. The miRNAs and the Ensembl ID of genes were directly entered into the databases respectively. The statistical values were automatically calculated and shown in the figure, mainly including the hazard ratio (HR) and log-rank p-value. Log-rank p-value < 0.05 was set as a threshold.

### Expression analysis of genes and lncRNAs

In GSE41258, 54 normal colon tissues, 186 CRC primary tumor lesions and 47 liver metastatic lesions were included in expression analysis of key genes. 186 primary CRC tumor tissues with clinical information grouped by tumor stage were incorporated into expression analysis of key genes. Likewise, in TCGA project, a total of 551 primary CRC tumor tissues with clinical data grouped by different tumor stages was included in expression analysis of predicted lncRNAs which could be related to prognosis in colorectal cancer metastasis. The expression analysis was visualized by the beeswarm package of R. P-value < 0.05 was regarded as statistically significant.

### Pearson correlation analysis

The ggplot2 and ggpubr packages of R were employed to visualize the Pearson correlation analysis of mRNA-miRNA, lncRNA-mRNA and miRNA-lncRNA pairs in CRC by using TCGA project. P-value < 0.05 was considered statistically significant.

### Sample collection

30 cases of colorectal cancer tissues and paired adjacent nontumor tissues were collected from patients with colorectal cancer from the Second Affiliated Hospital of Nanjing Medical University between 2019 and 2020. Tissues were frozen in liquid nitrogen and stored at -80° C. The experiment was approved by the Ethics Committee of the Second Affiliated Hospital of Nanjing Medical University, and informed consent was obtained from all patients. The clinical characteristics of 30 patients was shown in Supplementary Table 3.

### Cell culture and transfection

The human CRC cell lines HCT116 and SW480, purchased from the American Type Culture Collection (ATCC), were cultured in RPMI-1640 medium (Gibco, MD, USA) supplemented with 10% fetal bovine serum (FBS) (Gibco) and 1% penicillin/streptomycin (Gibco; Thermo Fisher Scientific, Inc.) in a humidified atmosphere of 5% CO_2_ at 37° C.

Small interfering RNAs (siRNAs) specifically targeting ZEB1-AS1 or APOC1, negative control siRNA (si-NC), miR-335-5p mimics and miR-335-5p inhibitor were all obtained from GenePharma Corporation (Shanghai, China). A plasmid vector, pcDNA-ZEB1-AS1 or pcDNA-APOC1, which could consistently express ZEB1-AS1 or APOC1, was constructed by GenePharma Corporation. Cell transfection of siRNA, miRNA mimic or inhibitor and plasmid vectors were all conducted with Hieff Trans™ Liposomal Transfection Reagent (Yeason, Shanghai, China) and Opti-MEM (Gibco) for 48h, according to the manufacturer’s protocols.

### RNA isolation and real-time quantitative PCR (RT-qPCR)

TRIzol® reagent (Invitrogen, Carlsbad, CA, USA) was used to extract the total RNA from the tissues and cells, and according to the manufacturer's instructions. The PrimeScript™ RT reagent kit (TaKaRa, Kyoto, Japan) and miRNA 1st Strand cDNA Synthesis Kit (Vazyme, Nanjing, Jiangsu, China) was used to reverse-transcribe RNA into cDNA. The quantitative RT-PCR was performed using SYBR Green Kit and miRNA Universal SYBR qPCR Master Mix (Vazyme) with StepOnePlus™ Real-Time PCR System (Thermo Fisher Scientific, Waltham, MA, USA), following the manufacturer's protocol. The relative expressions of genes were calculated using the 2^-ΔΔCT^ method. The primers were listed in Supplementary Table 4.

### Western blot analysis

Western blotting was performed as previously described [61]. The primary antibodies against APOC1 (1:1000, #ab198288; Abcam, USA) and GAPDH (1:2000, #5174; Cell Signaling Technology, Boston, MA, USA) were used to incubate the membranes. Goat anti-rabbit IgG (H&L) (1:1000, No. A0208; Beyotime, Shanghai, China) was used as a second antibody.

### Dual-luciferase reporter assay

The luciferase report vectors (ZEB1-AS1-WT / ZEB1-AS1-Mut and APOC1-WT / APOC1-Mut) were built from GenePharma Corporation (Shanghai, China). HCT116 cells were co-transfected with ZEB1-AS1-WT or ZEB1-AS1-Mut and miR-335-5p mimics or negative control (NC) using Hieff Trans™ Liposomal Transfection Reagent (Yeason, Shanghai, China) respectively. The cells were co-transfected with wild-type or mutated APOC1 constructs and miR-335-5p mimics or negative control (NC) using the same way as above. The Luciferase Reporter Assay System (Promega, Madison, WI, USA) was used to measure the relative luciferase activity in each group according to the manufacturer's instructions.

### Transwell migration and invasion assay

Cell transwell migration and invasion assays were performed using transwell chambers (8 μm PET; Millipore Corporation, Burlington, MA, USA). For the migration assay, cells (1×10^5^/ml) suspended in serum-free RPMI-1640 medium were seeded into the upper chamber. For the invasion assay, cells (1×10^5^/ml) suspended in serum-free RPMI-1640 medium were seeded into the upper chamber precoated with 0.5 mg/L Matrigel (BD Biosciences, Franklin Lakes, NJ, USA). Both of their lower chambers were filled with 800μL RPMI-1640 medium supplemented with 10% serum. The plates were incubated for 24 hours. The migrated or invaded cells were fixed with methanol for an hour at room temperature and stained with 0.4% crystal violet staining solution for 30 min at room temperature. The cells on the top of the membranes were removed with swabs, and then the remaining cells were washed twice with PBS. The migrated or invaded cells were counted in 5 random fields.

### Statistical analysis

Graphpad Prism (version 7.0) was applied to perform statistical analysis. The results are presented as mean ± SD at least 3 independent experiments. Statistical significance between groups was assessed by using Student's t-test, one-way ANOVA or χ^2^ test. P<0.05 was considered as statistically significant.

## Supplementary Material

Supplementary Tables
