# Supplementary material for: Construction of a novel mRNA-miRNA-lncRNA network and identification of potential regulatory axis associated with prognosis in colorectal cancer liver metastases
Source: Aging (Albany NY). 2021 Jun 3;13(11):14968–88. doi: 10.18632/aging.203049 (PMC8221294; doi:10.18632/aging.203049)
Supplement: Supplementary Tables [file aging-13-203049-s001.pdf]

SUPPLEMENTARY TABLES

Supplementary Table 1. Identification of 46 DEGs from datasets of CRLM.

DEGs	GSE41258		GSE49355		GSE68468		GSE81558	
	log_2FC	p-value	log_2FC	p-value	log_2FC	p-value	log_2FC	p-value
AHSG	2.80	<0.001	2.94	<0.001	2.60	<0.001	1.86	<0.001
ALDH8A1	2.26	<0.001	2.15	<0.001	2.02	<0.001	2.20	<0.001
APCS	1.82	<0.001	3.08	<0.001	2.51	<0.001	5.00	<0.001
APOA1	3.11	<0.001	2.98	<0.001	3.42	<0.001	3.21	<0.001
APOA2	2.88	<0.001	3.45	<0.001	3.65	<0.001	4.73	<0.001
APOB	2.20	<0.001	2.66	<0.001	2.59	<0.001	4.04	<0.001
APOC1	1.67	<0.001	3.01	<0.001	1.56	<0.001	2.06	<0.001
APOC3	4.91	<0.001	4.55	<0.001	5.03	<0.001	3.73	<0.001
APOH	3.99	<0.001	4.48	<0.001	3.65	<0.001	3.37	<0.001
C8A	1.56	<0.001	2.05	<0.001	2.24	<0.001	2.01	<0.001
C9	2.54	<0.001	2.97	<0.001	2.56	<0.001	3.62	<0.001
CFHR4	2.61	<0.001	1.90	0.0035	2.22	<0.001	2.83	<0.001
CP	1.67	<0.001	2.42	<0.001	2.11	<0.001	4.01	<0.001
CPB2	1.62	<0.001	2.47	<0.001	2.45	<0.001	2.89	<0.001
CYP2E1	1.77	<0.001	4.23	<0.001	3.08	<0.001	3.15	<0.001
F2	2.15	<0.001	2.61	<0.001	2.71	<0.001	1.67	<0.001
FBLN1	-1.85	<0.001	-1.58	<0.001	-2.00	<0.001	-1.94	<0.001
FGA	3.07	<0.001	3.11	<0.001	3.07	<0.001	5.37	<0.001
FGB	3.79	<0.001	4.47	<0.001	4.85	<0.001	5.40	<0.001
FGG	4.89	<0.001	5.31	<0.001	4.45	<0.001	4.63	<0.001
FGL1	1.82	<0.001	2.93	<0.001	2.04	<0.001	3.81	<0.001
GC	2.61	<0.001	4.89	<0.001	3.24	<0.001	3.30	<0.001
GREM1	-2.07	<0.001	-1.84	0.0037	-1.52	<0.001	-1.98	0.0012
HP	4.82	<0.001	4.75	<0.001	4.66	<0.001	5.41	<0.001
HPR	5.84	<0.001	5.57	<0.001	5.04	<0.001	2.24	<0.001
HPX	1.98	<0.001	2.99	<0.001	2.28	<0.001	2.81	<0.001
HRG	1.69	<0.001	2.52	<0.001	2.14	<0.001	3.17	<0.001
IGFBP1	2.45	<0.001	2.81	<0.001	2.89	<0.001	4.58	<0.001
ITIH2	1.82	<0.001	2.37	<0.001	1.65	<0.001	2.78	<0.001
ITIH3	2.06	<0.001	3.47	<0.001	2.64	<0.001	3.32	<0.001
MMP1	-3.64	<0.001	-4.59	<0.001	-4.00	<0.001	-1.98	0.0010
MMP2	-1.79	<0.001	-1.87	<0.001	-2.00	<0.001	-1.84	<0.001
MMP3	-2.98	<0.001	-4.11	<0.001	-2.96	<0.001	-2.14	<0.001
ORM1	4.80	<0.001	5.28	<0.001	5.52	<0.001	4.20	<0.001
ORM2	1.74	<0.001	4.86	<0.001	5.22	<0.001	3.72	<0.001
PLG	3.16	<0.001	3.01	<0.001	2.86	<0.001	2.31	<0.001
PLGLB1	1.65	<0.001	2.19	<0.001	1.56	<0.001	1.91	<0.001
PLGLB2	1.65	<0.001	2.19	<0.001	1.56	<0.001	2.04	<0.001
SAA1	1.67	<0.001	2.93	<0.001	2.15	<0.001	2.20	<0.001
SAA2	2.34	<0.001	2.93	<0.001	2.15	<0.001	2.80	<0.001
SERPINA1	1.81	<0.001	1.85	<0.001	1.78	<0.001	4.06	<0.001
SERPINA3	2.38	<0.001	3.70	<0.001	3.40	<0.001	4.43	<0.001
SERPINC1	1.93	<0.001	3.16	<0.001	2.59	<0.001	3.62	<0.001
TTR	2.51	<0.001	2.45	<0.001	2.32	<0.001	2.36	<0.001
VTN	2.02	<0.001	4.01	<0.001	3.95	<0.001	2.21	<0.001
VWF	-2.26	<0.001	-2.38	<0.001	-2.55	<0.001	-2.20	<0.001

Supplementary Table 2. Predicted upstream lncRNAs that may target miR-335-5p based on StarBase database.

miRNA	lncRNAs
miR-335-5p	RP11-429J17.2, TSPEAR-AS2, PAX8-AS1, LINC00943, SNHG17, CTBP1-AS2, MIR29B2CHG, TNRC6C-AS1, LINC00630, LINC01087, AP000320.1, DANCR, AP006222.1, XIST, RP11-284F21.7, AC021078.1, FTX, LINC01090, AC133785.1, AL590094.1, LINC01503, GAS5, SNHG20, LINC00941, ZEB1-AS1, AL627309.1, AL627309.3, AC106047.1, LINC00886, LINC00893, AC108488.4, NEAT1, UBR5-AS1, OIP5-AS1, TMEM161B-AS1, AL049795.2, THAP9-AS1, AC113615.1, TUG1, AF131215.4, AP003108.1, URB1-AS1, AC068888.1, AL132780.2, AC074091.1, AL357153.3, SSSCA1-AS1, AC009097.2, AL031123.2, AC007952.4, AC007342.5, AC016876.2, AC026271.3, AC012617.1, AC006504.5, RUNDC3A-AS1, AC007228.2 AC007228.9, KCNQ1OT1, SNHG8, AC007938.2, LPP-AS2, AC108463.3, AC093297.2, AC073335.2, PAXIP1-AS1, AC026356.2, RP11-305O6.4, AL161772.1, AC015871.3, AC005332.7, AC018628.1, AC092692.1, AC007191.1, AC108704.2, AC016542.2, MAPT-IT1, AP006248.3, AC022336.3, AC009108.5, PCBP3-OT1, LINC00294, SLFNL1-AS1, AC005394.2

Supplementary Table 3. Characteristic of 30 patients with colorectal cancer.

Characteristics	Case with metastasis (n = 16)	Case without metastasis (n = 14)	p-value
Age (Years)			0.873
<60	5	4	
≥60	11	10	
Gender			0.919
Male	10	9	
Female	6	5	
Location			0.295
Colon	13	9	
Rectum	3	5	
Tumor size			1.000
<5 cm	8	7	
≥5 cm	8	7	
Differentiation			0.050
Well / Moderate	10	13	
Poor	6	1	
Depth of tumor			0.022
T1+T2	0	4	
T3+T4	16	10	
Expression of APOC1			0.003
Low	4	11	
High	12	3	
Expression of ZEB1-AS1			0.028
Low	5	10	
High	11	4	
Expression of miR-335-5p			0.028
Low	11	4	
High	5	10	

Supplementary Table 4. Premiers for RT-qPCR.

ID	Sequence (5’ – 3’)
GAPDH-F	TCAACGGATTTGGTCGTATTG
GAPDH-R	TGGGTGGAATCATATTGGAAC
U6-F	CTCGCTTCGGCAGCACA
U6-R	AACGCTTCACGAATTTGCGT
ZEB1-AS1-F	CCAGACACCTACACAACTTCC
ZEB1-AS1-R	GTGATCTCTACCCTCTTGCTT
hsa-miR-335-5p-F	CGCGTCAAGAGCAATAACGAA
hsa-miR-335-5p-R	AGTGCAGGGTCCGAGGTATT
APOC1-F	ACCCACTT AGAGTTGTGAGCCC
APOC1-R	CAGACCACCTTAGTCCCTTTCC
